# International Society for Computational Biology Honors Dana Pe'er with Top Bioinformatics/Computational Biology Award for 2014

**DOI:** 10.1371/journal.pcbi.1003682

**Published:** 2014-06-19

**Authors:** Christiana N. Fogg, Diane E. Kovats

**Affiliations:** 1 Freelance Science Writer, Kensington, Maryland, United States of America; 2 International Society for Computational Biology, La Jolla, California, United States of America

The International Society for Computational Biology (ISCB) honors the achievements of an early- or mid-career scientist with the Overton Prize each year. The Overton Prize was established in memory of Dr. G. Christian Overton, a respected computational biologist and founding ISCB Board member who passed away unexpectedly in 2000. Winners of the Overton Prize are independent scientists in the early or middle phases of their careers that are recognized for their significant contributions to computational biology through research, teaching, and service. ISCB is thrilled to recognize Dr. Dana Pe'er (Image 1), Associate Professor in the Department of Biological Sciences and Systems Biology at Columbia University in New York, NY, as the 2014 winner of the Overton Prize. In recognition of this award, Dr. Pe'er will be a keynote speaker at this year's Intelligent Systems for Molecular Biology conference in Boston, Massachusetts and will present a talk titled “A Multidimensional Single Cell Approach to Understand Cellular Behavior” on Monday, July 14, 2014.

**Figure:**
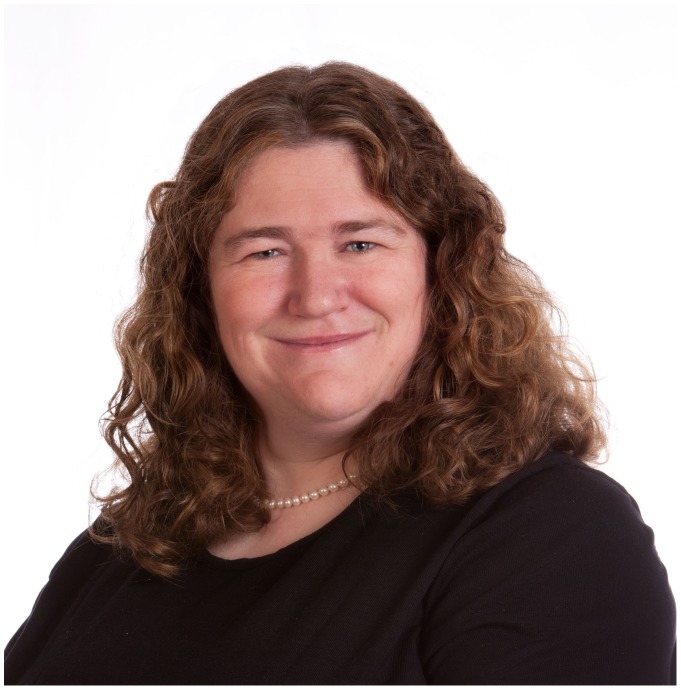
Image 1. Dana Pe'er. This image is credited to Vera LaMarche, American Association for Cancer Research.

## Dana Pe'er: From Mathematics to Mass Cytometry

Dana Pe'er encountered her first love in second grade. Her father was eager to instill a passion for learning in her, and one day he showed her the proof demonstrating why the same number of natural numbers and rational numbers exist, whereas the number of irrational numbers is greater than the number of rational numbers. Pe'er recalled, “Grappling with different strengths of infinity and the elegance [of] mathematical logic made me fall in love with math.”

Pe'er had her first taste of research while she was a high school student. She worked in the lab of Dr. Idan Segev at the Hebrew University of Jerusalem. This experience opened her eyes to the many patterns that exist in nature, and she recognized how “biology was in sore need of new mathematical approaches that can aid in the interpretation of biological phenomenon.” Pe'er used mathematical modeling in Segev's lab to study subthreshold oscillations in neurons, and this experience led her to consider studying neurobiology. But her path shifted toward computational biology after she heard a charismatic talk by Dr. Eric Lander at the Weizmann Institute about the emerging field of genomics.

Pe'er received her bachelor's degree in mathematics, as well as her master's and PhD degrees in computer science, from the Hebrew University of Jerusalem. She did her PhD research in the lab of Dr. Nir Friedman, where she had the realization that “statistical machine learning is a very powerful ‘math’ to help elucidate biology, and the complexity of it all required computer science.” She recalls gaining several insights during this period that have accompanied her throughout her career, including her affirmation, “Good modeling of the biology is the most important ingredient toward a good computational method for biological discovery. Rather than applying the most sophisticated, ‘nuclear-powered’ method to squeeze the most out of the data statistically, one can use biological insight to limit the space of possible models more than any statistical method ever can.” Making the right assumptions requires a good understanding of biology, knowledge Pe'er gained through her collaboration with Dr. Aviv Regev. They met as graduate students in Israel, where Regev greatly influenced how Pe'er thought of biological questions. Pe'er recalled, “She was my first real biology teacher, and she taught me to think about biology more abstractly rather than stick to more rigid and dogmatic thinking.”

Pe'er did her postdoctoral work with Dr. George Church at Harvard University, where she began navigating the messy world of experimental biology. Church's mentorship gave Pe'er a new perspective on science, and she moved away from asking, “What type of computation can I do for this data?” and learned to ask instead, “What data do I need to answer a biological question I am passionate about?”

ZIt is good to receive mentorship from many sources, and Pe'er describes the mentorship she received from Dr. Daphne Koller as being instrumental to her success as a trainee. Koller provided guidance and mentoring to Pe'er during her PhD and postdoctoral training and instilled in her the importance of “good modeling assumptions.” Although Pe'er was not an official student of Koller's, she recalls appreciatively the valuable career advice and insights Koller shared with her as she launched into her career as an independent researcher.

In 2006, Pe'er started her own lab at Columbia University in the Department of Biological Sciences and Systems Biology. Her lab embodies the interdisciplinary nature of her research and is filled with trainees from a wide range of backgrounds, including computer science, genetics, applied math, and biomedical engineering. She genuinely appreciates working with her trainees and has enjoyed “watching them grow, and seeing how much they matured as scientists.” She stated, “I really love mentorship and feel a form of motherhood towards my trainees.”

Pe'er has developed several research projects that use large, complex datasets to examine how molecular networks respond to various external stimuli. One of her primary interests is using single cell technologies such as mass cytometry to better understand cellular heterogeneity. She is fascinated by this work and hopes “to reframe development not as a set of discrete cell types, but rather as a continuum, a dynamic process in which one can place each individual cell along a developmental trajectory that represents not only cell types, but their many intermediates.” Pe'er is planning to apply her studies of cellular heterogeneity to cancer and the improvement of personalized cancer therapy. She sees the single-cell approach as a powerful tool to identify and target treatment-resistant tumor cells.

Pe'er has always let the data steer her toward new areas of study. “I let the data itself guide me, combining [with that] biological knowledge, yet an open mind,” she explained, continuing, “I look for patterns and structure and expect the unexpected. Exploring complex data is lots of fun and requires looking at the data, playing with it, getting a feel for it.” But she also wrestles with the challenges of using biology to guide her modeling assumptions. “A good biological assumption can really shape a good model and limit the search space when fitting data. But a bad biological assumption can set you off on the wrong path,” she warned. “Math is rigorous, and biology is messy, so the trick is to find the pattern in the mess, and machine learning provides a powerful toolbox.”

Pe'er's training in computer science and biology have given her a unique combination of skills and knowledge that have served her well as a computational biologist, but she predicts that her training experience will become mandatory for future biology researchers. As she sees it, “Biology has become an information science. Enabled by an increasing number of technologies, the magnitude and complexity of the data is only increasing. In the future, computation will be an integral part of biology, like molecular biology is today.” In the meantime, Pe'er champions the power of doing science at the interface of biology and computation. “My ‘bilingual’ training really lets me play at the interface,” she acknowledges. “It lets me communicate with both sides effectively and make connections. By understanding what powerful computation can do, I can design experiments and strive for technologies that might not be intuitive and obvious to a bench biologist that is less versed in computation. Designing the right data-rich experiment, matched with the right biology, is truly empowering.”

Pe'er serves on the editorial board of the journal *Cell*, and she considers this role a valuable opportunity to serve the scientific community. *Cell* has acknowledged computational biology as a critical rising field, and Pe'er sees her work on the advisory board as an important way to serve the computational biology community and help educate the journal about the field.

Outside of the lab, Pe'er has taken time to support and promote K12 science education by organizing a science expo. She was inspired to do this when she realized that her young daughter didn't really know what she did. Pe'er recalled that her daughter “thought [her] job was ‘writing emails all day.’” She continued, “She did not realize that scientists are trying to figure out what we don't know, rather than rehash what we do.” The expo was designed to transform a school into “a multi-story, hands-on, interactive science museum.” “Each volunteer scientist brings their lab and science to the kids, distilled in a way that is both engaging and clear to the kids,” Pe'er explained. She acknowledged that the expo presents a big but gratifying challenge to the volunteer scientists because they had to take “complex science and distill it in a way that can relate to a five-year-old.” “But if you can explain your science to a five-year-old, you can explain it to anyone,” she pointed out.

Dr. Alfonso Valencia, chair of the ISCB Awards Committee, sees Pe'er's selection as fitting recognition of her scientific contributions. He said, “I was very happy to see that Dana Pe'er was finally selected for the award. This is always a very difficult decision, given the number of excellent young computational biologists in our community. Dana has published amazing papers with substantial impact in biology and cancer biology, together with other papers on method development that were very influential, some of them presented in the ISMB [Intelligent Systems for Molecular Biology] conference.” Dr. Bonnie Berger, co-chair of the Awards Committee, also sees Pe'er as a rising luminary in the field of computational biology, “for pioneering the use of Bayesian networks in cellular network inference.”

Pe'er is looking forward to where her research will take her, especially her ongoing work on single cell data. She is also enjoying and appreciating this moment of recognition. Pe'er was excited and uplifted when she was told she had been selected for the 2014 Overton Prize, and she recounted, “I got such an outpouring of congratulations from mycolleagues, which was really the best.”

